# The impact of digital governance on the health of rural residents: the mediating role of governance efficiency and access to information

**DOI:** 10.3389/fpubh.2024.1419629

**Published:** 2024-07-26

**Authors:** Yongzhou Chen, Qiuzhi Ye

**Affiliations:** ^1^School of Public Policy and Management, Guangxi University, Nanning, China; ^2^Institute of Green Development and Borderland Governance, Guangxi University, Nanning, China; ^3^Institute of Strategic Development, Guangxi University, Nanning, China

**Keywords:** digital governance, health, rural governance, information access, longevity, mental health

## Abstract

**Background:**

Digital transformation in rural areas has become a key policy priority worldwide. China is also implementing a digital village strategy and actively promoting the digital transformation of rural governance to improve the well-being of rural residents. The literature suggests that digital governance is linked to health, but the mechanisms behind this relationship remain unclear.

**Methods:**

Using data from the 2021 China Land Economic Survey (CLES), this paper examines the impact of digital governance on the health, longevity, and mental health of rural residents. To enhance the robustness of the conclusions, this paper also introduces a dual machine learning model to solve the endogeneity problem of the model.

**Conclusion and discussion:**

This study concludes that digital governance has a significant positive impact on the health of rural residents. This finding remains consistent even after addressing endogeneity issues and conducting numerous robustness tests. Mechanistic analyses indicate that digital governance can enhance rural residents’ health by improving village governance (environmental governance) and increasing the efficiency of access to personal information. Further analysis reveals that digital governance significantly increases the life expectancy of rural residents but that its effect on mental health is not significant. This study provides new insights into how digital governance affects health, with important implications for health policy development.

## 1 Introduction

In numerous countries, rural populations experience health inequities because of insufficient health facilities and services in rural areas (1–3). Similarly, there is also a huge urban–rural health gap in China, with rural residents generally experiencing poorer health than their urban counterparts (4). In response, the Chinese government has implemented robust policy initiatives to address this issue. For instance, the integration of urban–rural healthcare, an inclusive and equitable health policy, has significantly improved rural residents’ health (5). The Chinese government’s “rural digital governance” program may also positively impact rural health. Research has shown that using digital technologies can help improve rural health (6–10). This suggests that digital governance in rural areas may have a positive health impact. However, the current research is only at the theoretical level and lacks empirical evidence to support this conclusion. Therefore, it is important to study this impact in the context of the accelerating global evolution of digital governance. This will help in fully understanding the health impacts of digital transformation.

A global development policy focuses on digitalizing rural areas (11). The development of data science and artificial intelligence technology is fueling the emergence of the third wave of digital governance. This will lead to more efficient and intelligent governance (12). China is promoting the digital transformation of governance and has achieved positive results (13). Digital governance has emerged as a significant policy element in rural China. For instance, the Action Plan for the Development of Digital Rural Areas (2022–2025) emphasises the necessity to enhance digital governance capacity, improve the system of intelligent party building in rural areas, and extend “Internet + government services” to the countryside (14). In the realm of technology-driven governance, rural digital governance can be conceptualized as the employment of digital intelligence technologies to establish a digital framework for overseeing and managing rural affairs. This includes creating a comprehensive information infrastructure and governance module, transforming the production and processing of governance information, and designing a “holistic and intelligent” form of governance on this foundation (15). Empirical evidence indicates that the absence of effective governance is a significant obstacle to enhancing public health. The utilisation of innovative digital approaches can prove beneficial in the promotion of good governance within the health sector of low- and middle-income countries (16), and many studies have suggested and examined digital governance frameworks for health promotion (17–20). From this literature, it can be inferred that the implementation of digital governance in rural China may affect related factors to improve health, such as the efficiency of rural governance (particularly environmental governance) (21) and the availability of sufficient medical information. It is crucial to ascertain whether the aforementioned potential associations are indeed present, as this will facilitate the generation of novel insights into the influence of digital governance on health.

However, less attention is given to the impact of digital governance on health, despite a large body of literature exploring the factors that influence the health of rural residents. The determinants of health can be classified into two principal groups: those that operate within the individual and those that act externally. Internal factors refer to individual characteristics and capabilities, such as demographics (22), health behaviors (23), health literacy (24), and deprivation (25). External factors refer to policy and environmental variables such as resource availability (26), social protection (27), health services (28), social capital (29), culture (30), housing, and discrimination (31). A review of the literature does not reveal any evidence on how digital governance affects health. Therefore, further research on this topic is needed. We utilise survey data [the China Land Economic Survey (CLES)] to explore this issue and, later, to validate the theoretical hypotheses and provide new insights into the health effects of digital governance.

The marginal contribution of this study compared to established studies is as follows: First, there is a paucity of research examining the effect of digital governance on the health of rural residents. However, China is actively implementing a programme to enhance rural governance capacity in the areas of the economy, environment, and health. It is therefore of the utmost importance to gain an understanding of the interrelation between digital governance and population health in global digital transformation. Second, this study examines the effect of digital governance on health, specifically in terms of improved governance and access to information. Additionally, a machine learning approach is introduced to solve the endogeneity problem, further enhancing the credibility of the conclusions.

The paper is structured as follows: The theoretical hypotheses section proposes three research hypotheses. The data and methods section describes the data sources, variable selection, and model setting. The section on empirical results analyzes the direct and indirect effects of digital governance on the health, including longevity and mental health. The final section presents conclusions and recommendations.

## 2 Theoretical assumptions

### 2.1 The effect of digital governance on the health of rural residents

In theory, digital governance has many advantages. It can expand channels, share information, and improve governance, which can benefit the health of rural residents. The channel effect of digital governance is the most significant advantage. Digital governance relies on the extensibility of platforms. It can continuously integrate and link numerous functions, providing new channels for accessing health services. For instance, some rural villages in Jiangsu, China, have integrated health care services, including medical care, older adult care, and medical check-ups, onto digital platforms, providing online access to residents. Furthermore, the development of industries such as telemedicine and smart health care, supported by digital technology, has made health care services more convenient and accessible for rural groups (32). However, it is important to acknowledge that digital governance is a distinct concept from telemedicine. Telemedicine is a term used to describe the use of internet information technology to provide medical information and services to patients in remote locations or in environments where traditional medical care is not readily available. It is typical for these services to be excluded from the provision of public goods and to be more closely aligned with commercial medical services. The current lack of widespread availability of telemedicine in rural China is largely attributed to the high operating costs associated with its implementation. Telemedicine is therefore clearly distinct from basic public healthcare services delivered through digital governance. The theory of industrial clusters posits that the concentration of related industries can result in a reduction of institutional costs for firms and an increase in economies of scale. Similarly, investing in digital health care in areas with a favorable digital environment, including sufficient digital literacy and infrastructure, can reduce construction and operating costs. Therefore, digital health investors will prefer locations with good digital governance. In summary, digital governance can assist rural residents in overcoming physical space constraints to access and enjoy health services, to the benefit of their personal health.

Second, digital governance facilitates information-sharing. The central component of digital governance is data governance. The use of digital intelligence technologies can improve data visualization and sharing, leading to increased information efficiency in rural areas (33). According to the theory of information visualization, presenting complex information through graphics and videos can improve audience acceptance (34). Therefore, digital governance can enable rural populations to access the information they need for their health, either directly or indirectly. In addition, the “Internet + government platform” in villages has been equipped with a range of health services for residents’ benefit. For instance, Tangxi Village in Jiangsu Province utilizes digital governance to offer health services to its residents. This includes the provision of health bracelets to older adult people, intelligent water quality monitoring, and intelligent medical care. Therefore, concerning rural health, digital governance can enable analysis of various behaviors, indicators, and other information; monitor the health of rural residents; and provide timely information on potential health risks to rural doctors or residents themselves.

Third, digital governance can enhance the efficiency of traditional governance through technology. From a technical perspective, it involves relying on digital platforms to decentralize and transform the governance structure (35). This process enables more individuals to participate equally, thereby increasing participation in rural governance by multiple parties (36). Coordination and cooperation can be more difficult in the absence of a rational structure and adequate communication among stakeholders, based on the organizational structure and perspective. Digital governance can create a virtual digital space with a flat structure, transmit information in real time, and connect different stakeholders (37). This, in turn, can reduce the cost of coordination and improve governance capacity. Digital governance has clear advantages in environmental governance and infectious disease control, particularly in relation to rural health (21, 38). In light of the aforementioned findings, we propose the following hypothesis:

**Hypothesis H1**: The health of rural residents will be improved by digital governance.

### 2.2 The mechanisms through which digital governance affects rural residents’ health

The decline of rural areas presents numerous threats to residents’ health, including mental health, life expectancy, and neonatal mortality (39). Effective governance based on technological and institutional innovation is crucial for mitigating rural decline and achieving sustainable rural development (40). In general, good governance has a positive impact on villages’ economic development, the environment (including water, air, soil, etc.), and the provision of public services. This, in turn, is closely related to public health. With the help of digital intelligence technologies, digital governance can reconfigure rural partnerships (41) and significantly improve rural governance efficiency (42), which can, in turn, have a positive impact on rural health. Digital governance has been shown to significantly improve rural environmental management (21). Studies have also demonstrated that enhancing the village environment can lead to better health outcomes for rural residents (43–45). For instance, traditional latrine pits may indirectly impact residents’ health by contaminating drinking water sources (43). Therefore, digital governance is likely to improve rural health through enhanced environmental governance. This paper proposes the following hypothesis:

**Hypothesis H2**: Digital governance has the potential to enhance village governance, particularly environmental governance, which subsequently enhances the health of rural residents.

Theoretically, information can significantly improve public service delivery in rural areas. For instance, public health care can reduce overall morbidity (46). According to one study (47), rural populations have less opportunity to access health information from sources such as primary care doctors, specialists, journals, and search engines than their urban counterparts. This lack of access contributes to increased health problems among rural residents. However, rural digital governance can significantly facilitate the spread of information in rural areas, especially by expanding individuals’ access to information. Furthermore, digital governance in rural areas can increase the digital literacy levels among rural communities as well as expand the digital user base by way of digital feedback (48). The use of smart medical devices can provide health information directly to rural residents and reduce information asymmetry. Therefore, digital governance can increase rural residents’ access to information, particularly through the internet. In summary, we again propose the following hypothesis:

**Hypothesis H3**: Digital governance can enhance rural people's access to information, thereby improving their health.

**Figure 1:**
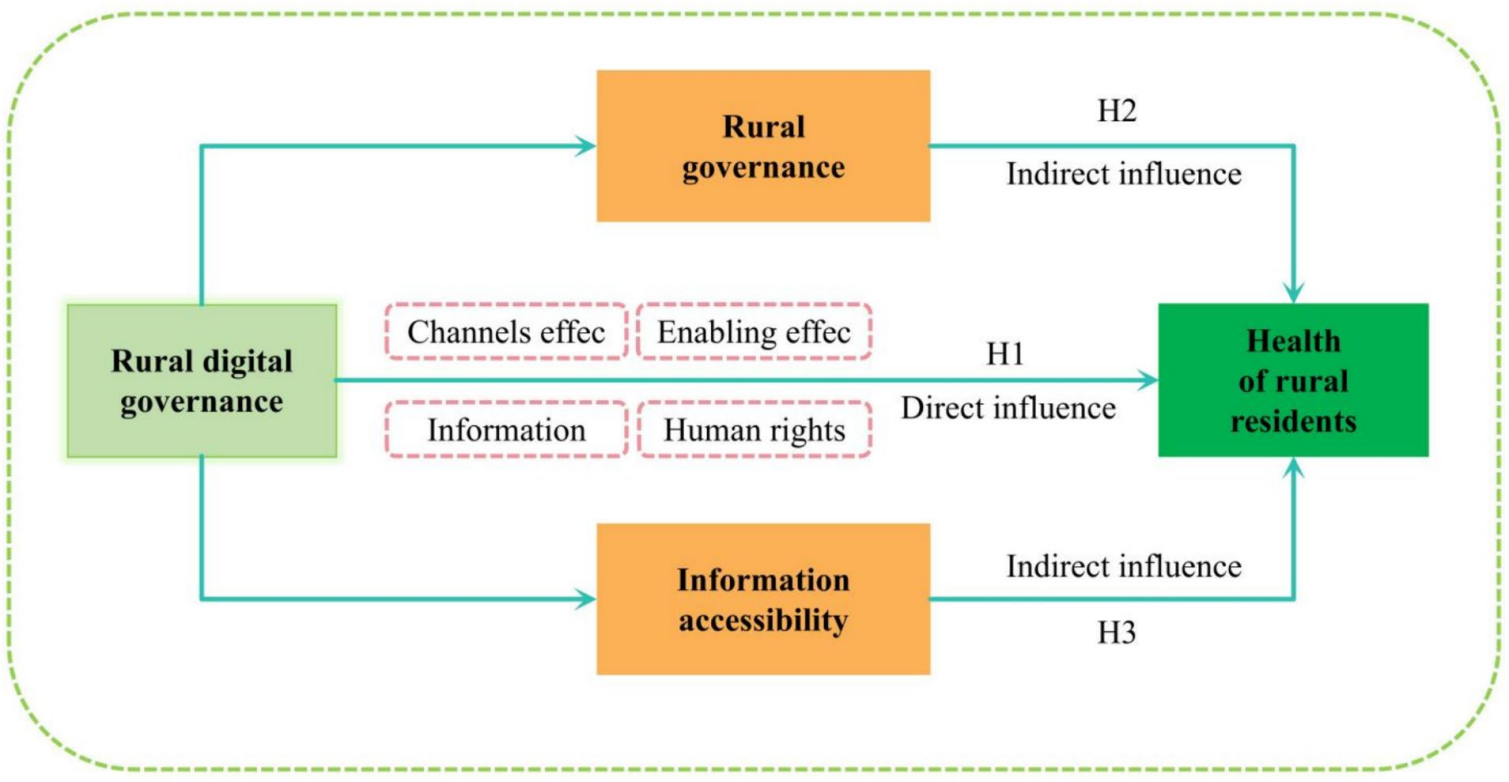
Mechanisms of influence between digital governance and the health.

Based on the above theoretical analysis and research hypotheses, the research framework of this paper is shown in Figure 1.

## 3 Data and methodology

### 3.1 Data

The paper utilizes data from the 2021 CLES, which was implemented by Nanjing Agricultural University. The survey employed probability proportional to size (PPS) sampling. A total of 26 counties and districts were randomly selected from 13 prefecture-level cities in Jiangsu Province. Additionally, 2 villages were randomly selected from each county and district, resulting in a sample of 52 administrative villages and 2,628 farm households. The survey questionnaire comprised two distinct sections: a Farm Household Questionnaire and a Village Questionnaire. The Farm Household Questionnaire primarily included questions on households’ production, land, health, income, assets, finances, and attitudes. The Village Questionnaire primarily encompassed demographic, economic, environmental, and infrastructural aspects of the villages. This paper removes samples with missing core variables, resulting in 2,292 valid samples.

Regarding the representativeness and appropriateness of the sample, Jiangsu, located in China’s southeastern coastal region, is a large agricultural province with a developed economy and a concentrated population. Geographically, Jiangsu can be divided into three regions: southern, central, and northern. North Jiangsu’s economy has seen relatively weak development, similar to those of rural areas in less-developed areas of China. Conversely, the central and southern regions exhibit a higher level of economic development, comparable to that observed in rural areas of developed regions. Jiangsu has more dramatic variations in rural digital governance. According to the County Digital Rural Index (2020) released by the Institute of New Rural Development of Peking University, Jiangsu’s average level of rural digital governance is 52.72, with a standard deviation of 15.13. This is higher than the national average for the same period, which is 48.54, with a standard deviation of 18.47. Overall, rural digital governance in Jiangsu is greater than the national average. However, there are still significant differences within the province, similar to the national situation. Therefore, research conclusions based on survey data in Jiangsu can provide a valuable reference for other regions.

### 3.2 Defining variables

#### 3.2.1 Dependent variable

The dependent variable of the paper is the level of health of the rural population. To measure the health of the population, we use respondents’ self-reported health, following related studies (49, 50), which is denoted as ***Health***. The measurement scheme is consistent with most studies and is the simplest and most accessible form of sampling. The questionnaire asked respondents and their family members about their self-perceived health status on a scale of 1 to 5, indicating low to high health. We also take the average of the self-reported health levels of the household members to calculate the average health level of the household (health_f), which is used for robustness testing. Additionally, we examine whether digital governance contributes to longevity and mental health, which are crucial aspects of health. Life expectancy is measured by analyzing the average age of individuals who passed away in the village during the current year. To measure mental health, we utilize the total score of the depression scale, drawing on relevant studies (51). Further analysis will cover these topics.

#### 3.2.2 Core explanatory variables

Rural digital governance is the explanatory variable of this paper. Since 2018, China has been implementing a digital countryside strategy that encompasses a range of digital elements, including digital foundations, the digital industry, digital governance, digital literacy, and other aspects. Rural digital governance is primarily based on “Internet + Party Building,” “Internet + Public Services,” and “Internet + Information.” The Central Internet Information Office, along with other departments, has jointly issued the Notice on Carrying out National Pilot Work on Digital Countryside and the Action Program for Digital Countryside Development (2022–2025). Both documents emphasize the need to extend “Internet + Party Building” and “Internet + Public Service” to rural areas to accelerate digital governance. The platform derived from “Internet +” is a crucial tool because it directly reflects the level of digital governance. We refer to the study (21), which measured digital governance using a village questionnaire. The questionnaire asked village council members about the development of digital governance. The options presented to respondents included four choices: “Internet + party building,” “Internet + government services,” “Internet + information management,” and “none of the above.” In this study, a village that had developed both “Internet + Party Building” and “Internet + Government Services” is assigned a digital governance level of 2. If a village had developed only one of the two, it is assigned a level of 1. If a village had not developed either of the relevant digital platforms, it is assigned a level of 0. To ensure the robustness of the results, we also remeasure villages’ digital governance (RDG_r) by taking into account “Internet + information management.” Villages are given a score of 1 if they met one of the conditions, and the level of digital governance is divided into four levels ranging from 0 to 3.

#### 3.2.3 Mediating variables

This paper considers the mediating variables of governance level (Gov) and information accessibility (IA). In terms governance, village governance is crucial for the village environment, public services, and economic development, which are closely related to public well-being. The CLES household questionnaire asked villagers to rate the effectiveness of governance in their village on a scale of 1 to 5, with larger scores reflecting better governance. We use this data to measure the level of rural governance. Based on the theoretical analysis above, this pathway is more likely to directly manifest in improved environmental governance (EGov), which in turned impacts the heath of the rural population. Garbage classification is an important strategy used by the Chinese government to promote environmental governance and improve the village environment. Garbage classification is a crucial aspect of public participation in environmental governance. This paper uses villagers’ garbage sorting behavior as a proxy variable for villages’ level of environmental governance. The CLES household questionnaire asked villagers whether they sorted their garbage, with 1 indicating yes and 0 indicating no.

Second, access to information can significantly impact residents’ health behaviors and decisions. This information should be presented objectively and without bias. In the digital age, rural residents have access to more appropriate information through online channels. The CLES household questionnaire surveyed respondents on their access to information using a scale ranging from 1 to 5. A score of 1 indicated access to information from non-Internet sources, while a score of 5 indicated access mainly from internet sources. Larger values on the scale indicated a greater proportion of access to information from internet sources. This paper uses these data to measure the population’s information access.

#### 3.2.4 Control variables

This paper considers 11 control variables at the individual, household, village, and regional levels based on data availability. The individual-level variables include the respondents’ gender (Gender), age (Age), and years of education (Edu). Household-level variables include *per capita* household income (PCHI), family medical resources (FMR), and family housing conditions (FHC). PCHI measures the income per person in a household, FMR measures whether or not a family has health insurance, and FHC measures whether or not a family’s home is in disrepair. The variables at the village level include the village economic level (VEL), which is determined by the income *per capita*; the village geographic location, which is indicated by the distance to townships (Distance) and topographic features (TF); and the village sanitation facilities (VSF), which is measured by the number of garbage cans *per capita*. At the regional level, this paper employs dummy variables for the three regions of southern, central, and northern Jiangsu to eliminate the potential influence of regional economic development levels and policies. See Table 1 for basic information about the above variables.

**Table 1:** Descriptive statistics for the variables.

Variable	Code	Obs	Mean	SD	Min	Max
Rural resident health	Health	2,292	4.050	1.049	1	5
Average family health level	Health_f	2,292	4.217	0.811	1	5
Rural resident longevity	longevity	2,292	73.59	9.099	28	87
Mental health	Mhealth	2,292	14.350	2.256	8	26
Rural digital governance	RDG	2,292	1.487	0.707	0	2
	RDG_r	2,292	2.119	1.018	0	3
Rural governance	Gov	2,292	4.149	0.799	0	5
Environmental governance	EGov	2,292	0.528	0.499	0	1
Information accessibility	IA	2,292	1.959	1.425	0	5
Gender	Gender	2,292	0.723	0.447	0	1
Age	Age	2,292	62.120	11.510	18	92
Educational level	Edu	2,292	7.179	3.991	0	19
*Per capita* household income	PCHI	2,292	9,855	19,719	0	134,000
Family Medical Resources	FMR	2,292	0.930	0.255	0	1
Family housing conditions	FHC	2,292	0.230	0.421	0	1
Village economic level	VEL	2,292	24,209	10,707	2,356	50,000
Distance to township	Distance	2,292	6.201	5.854	1	40
Topographic features	TF	2,292	1.155	0.362	1	2
Village sanitation facilities	VSF	2,292	0.143	0.186	0.001	0.769
Regional variables	Region	2,292	1.818	0.673	1	3

### 3.3 Modeling

The health level in this study is represented by ordered discrete data, making the ordered probit model the appropriate choice. The model is set as follows:

(1) $$\mathrm{Healt}{h_{i}}^{*}=\theta_{0}RDG_{i}+\mathrm{\beta Contro}l_{i}+\mathrm{Regio}n_{i}+\varepsilon i$$

(2) $$\mathrm{Healt}h_{i}=\left\{\begin{array}{l} 1,\mathrm{Healt}{h_{i}}^{*}\leq \mu_{1}\\ 2,\mu_{1}<\mathrm{Healt}{h_{i}}^{*}\leq \mu_{2}\\ 3,\mu_{2}<\mathrm{Healt}{h_{i}}^{*}\leq \mu_{3}\\ 4,\mu_{3}<\mathrm{Healt}{h_{i}}^{*}\leq \mu_{4}\\ 5,\mu_{4}<\mathrm{Healt}{h_{i}}^{*} \end{array}\right\}$$

Equation (1) uses the subscript *i* to denote the sample of household *i*. *Health_i_* represents the health level of rural residents, which is measured on a scale of five levels, with higher numbers indicating higher health levels. The model uses *RDG_i_* as the main explanatory variable to represent the level of rural digital governance. *Control_i_* refers to the set of control variables. *Region_i_* is a regional dummy variable that distinguishes among northern, central, and southern Jiangsu. The coefficients to be estimated are θ and β , and the random disturbance term is represented by $\varepsilon_{i}$ . Equation (2) defines ${\mathrm{Health}}_{i}$ as the level of health of the rural population, represented by an ordered variable ranging from 1 to 5.

To test Hypotheses H2 and H3, this paper constructs a mediation effect model based on stepwise testing.

(3) $$\mathrm{Me}{d_{i}}^{*}=\alpha RDG_{i}+\mathrm{\beta Contro}l_{i}+\mathrm{Regio}n_{i}+\varepsilon_{i}$$

(4) $$\mathrm{Healt}{h_{i}}^{*}=\theta_{1}RDG_{i}+\eta Med_{i}+\mathrm{\beta Contro}l_{i}+\mathrm{Regio}n_{i}+\varepsilon_{i}$$

In Equations (3) and (4), *Med_i_* represents the mediating variable, while the other symbols have the same meaning as in Equation (1). We first run a regression analysis on Equation (2). If α passes the significance test, there is an effect of digital governance on the mediating variable. Next, we estimate Equation (3), and if η passes the significance test, it indicates the existence of a mechanism of action whereby the digital governance pass-through mediator variable affects rural residents’ health.

## 4 Empirical results

### 4.1 Direct effects analysis

First, a test is run before the regression to prevent covariance. The results indicate that there is no significant multicollinearity problem in the model, as the variance inflation factor (VIF) has a mean value of 1.34 and a maximum value of 2.08, both of which are less than 10. Second, different levels of control variables are sequentially added to observe the changes in the model. Finally, to improve the precision of the results, cluster-robust standard errors (clustered to farmers) are used for estimation. Table 2 reports the results of the models.

**Table 2:** Baseline regression results for direct effects.

Variables	Health	Health	Health	Health
	Model 1	Model 2	Model 3	Model 4
RDG	0.126^***^ (0.032)	0.098^***^ (0.033)	0.103^***^ (0.033)	0.095^***^ (0.034)
Gender		0.049 (0.057)	0.052 (0.057)	0.031 (0.058)
Age		−0.026^***^ (0.003)	−0.026^***^ (0.003)	−0.025^***^ (0.003)
Edu		0.046^***^ (0.007)	0.042^***^ (0.007)	0.038^***^ (0.007)
PCHI			<0.001 (<0.001)	<0.001 (<0.001)
FMR			−0.024 (0.091)	−0.029 (0.092)
FHC			−0.326^***^ (0.054)	−0.317^***^ (0.055)
VEL				<0.001^***^ (<0.001)
Distance				0.008^*^ (0.005)
TF				−0.121^*^ (0.074)
VSF				0.380^**^ (0.152)
Region	NO	NO	NO	YES
Log likelihood	−2957.677	−2815.285	−2796.743	−2775.370
Wald	15.43^***^	307.62^***^	348.09^***^	366.72^***^
Pseudo R^2^	0.003	0.051	0.057	0.064
Obs	2,292	2,292	2,292	2,292

The symbols ***, **, and * indicate significance levels of 1, 5, and 10%, respectively. Clustering-robust standard errors are shown in parentheses.

As shown, all the Wald tests indicate a significance level of 1%, which suggests that the model is valid. Model 1 does not include any control variables. The results indicate that digital governance significantly improved the health of rural residents. Models 2, 3, and 4 demonstrate that the coefficients of digital governance remain stable and continue to have a beneficial impact upon rural population health, even after the gradual introduction of control variables at the individual, household, village, and regional levels. The digital transformation of rural governance has brought digital dividends to rural residents. Specifically, digital governance can effectively improve their health. Hypothesis H1 is verified.

Model 4 presents information on the control variables. First, health decreases significantly with age. Second, a longer education duration leads to improved health. Third, family housing conditions have a significant impact on the health of rural residents. Dilapidated family housing significantly reduces rural health. Finally, raising the economic level and upgrading the health infrastructure of a village can significantly enhance the health status of its residents.

### 4.2 Indirect effects analysis

The above results demonstrate that digital governance can significantly improve rural residents’ health. This section examines the mechanism of its impact to further test Hypotheses H2 and H3. Table 3 presents the mediated effect estimates.

**Table 3:** Regression results for impact mechanisms - indirect effects.

Variables	Improve the governance level				Improve the information accessibility	
	Gov	Health	EGov	Health	IA	Health
	Model 1	Model 2	Model 3	Model 4	Model 5	Model 6
RDG	0.109^***^ (0.034)	0.085^**^ (0.034)	0.120^***^ (0.041)	0.089^***^ (0.034)	0.179^***^ (0.034)	0.088^***^ (0.034)
Gov		0.130^***^ (0.032)				
EGov				0.117^**^ (0.049)		
IA						0.041^**^ (0.021)
Controls	YES	YES	YES	YES	YES	YES
Region	YES	YES	YES	YES	YES	YES
Log likelihood	−2508.372	−2765.830	−1444.608	−2772.555	−3033.325	−2773.316
Wald	71.37^***^	388.23^***^	228.33^***^	373.71^***^	688.71^***^	375.98^***^
Pseudo R^2^	0.015	0.067	0.089	0.065	0.114	0.065
Obs	2,292	2,292	2,292	2,292	2,292	2,292

The symbols *** and ** indicate significance levels of 1, 5, and 10%, respectively. Clustering-robust standard errors are shown in parentheses.

The results in indicate that digital governance performs a significantly positive regression coefficient on the degree of governance at the 1% level. This suggests that digital governance improves governance in villages. Model 2 demonstrates that digital governance and the level of governance have a significant positive impact on the health of rural residents. These findings suggest that digital governance can enhance rural health by improving governance. The mediating effect of village governance level is supported, partially confirming Hypothesis H2. Similarly, the results of Model 3 indicate that the regression coefficient of digital governance on the level of environmental governance is significant and positive at the 1% level. This suggests that digital governance improves environmental governance in villages. Model 4 demonstrates that digital governance and environmental governance both have a significant positive effect on the health of rural inhabitants. Therefore, we can conclude that digital governance improves environmental governance, which in turn improves the health of the rural population. The mediating effect of environmental governance also holds, supporting Hypothesis H2.

Prior to the analysis of the regression model, this paper presents some data to illustrate the increase in farmers’ access to health information following the implementation of digital governance. As the data employed in this study is cross-sectional in nature, To validate this, a comparison is made between villages that have not undergone digital governance transformation (RDG = 0) and villages that have undertaken digital transformation (RDG ≥ 1). Using information on the use of online forms of insurance payment by farm households, this paper calculates the proportion of health insurance premiums paid online by each household (PHIPPO). The utilisation of online forms to facilitate the payment of health insurance premiums reflects both the digital literacy of farmers and their enhanced understanding of the processes involved in accessing health services. Consequently, the comparison of this data allows for a more comprehensive understanding of whether there are disparities in the accessibility of health information among rural residents in areas with varying levels of rural digital governance. The data indicates that in villages where digital governance is not implemented (RDG = 0), PHIPPO is only 2.184 per cent. Upon reaching RDG = 1, PHIPPO ascends to 7.456 per cent. Upon reaching RDG = 2, PHIPPO further increases to 8.163 per cent. The data indicates that the implementation of rural digital governance has a positive impact on the health information and digital literacy of farmers. Table 3 Model 5 demonstrates that digital governance significantly enhances rural residents’ access to information. Model 6 indicates that the regression coefficients of both digital governance and access to information are significantly positive, suggesting that digital governance and access to information significantly enhance the health of rural residents. Taken together, these findings establish the mediating role of information access. This indicates that digital governance significantly enhances individual rural residents’ access to information, thereby contributing to improved personal health. Hypothesis H3 is supported.

### 4.3 Robustness testing

#### 4.3.1 Endogenous treatment

The above is a preliminary validation of the potential of digital governance to promote better health. However, the model may potentially be endogenous. To address this issue, this paper employs a dual machine learning approach and an instrumental variables approach. Traditional regression models are prone to estimation bias due to nonlinear relationships among variables and the ‘curse of dimensionality’ caused by too many covariates (52). To address endogeneity, we employ a dual machine learning model. Scholars have turned to machine learning for its advantages and applications in causal inference (53, 54), with dual machine learning being a typical example (52). For instance, Yang et al. (55) utilized a gradient boosting model in dual machine learning to reassess the influence of an expert auditor (a Big N auditor) on a company’s audit quality. They demonstrated that this approach is more resilient than the propensity matching method (55). The dual machine learning models are constructed based on semiparametric models, which are theoretically not endogenous. Therefore, if digital governance still significantly improves the health of rural residents after estimation using this model, endogenous interference can be ruled out. Following the research (52), we establish the following dual machine learning model:

(5) $$\mathrm{Healt}h_{i}=\theta_{2}RDG_{i}+g\left(X_{i}\right)+U_{i},E\left(U_{i}\;|\;X_{i}\;|RDG_{i}\right)=0$$

In Equation (5), subscript *i* denotes the sample of household *i*. *Health* is the dependent variable, while *RDG* is the explanatory variable for digital governance. The coefficient of disposition $\theta_{2}$ indicates the impact of digital governance on the health of the rural population; $X_{i}$ is the set of high-dimensional control variables that affect the health of rural residents through a function $g\left(X_{i}\right)$ . However, the exact form of the object is unknown, and its estimate, $\hat{g}\left(X_{i}\right)$ , can be obtained through machine learning methods. The error term, $U_{i}$ , satisfies the assumption of having a zero mean.

Equation (5) may experience slow convergence. To expedite convergence and ensure unbiased estimates, we also construct the following auxiliary regressions:

(6) $$RDG_{i}=m\left(X_{i}\right)+V_{i},E\left(V_{i}|X_{i}\right)=0$$

where $m\left(X_{\mathrm{it}}\right)$ represents the regression function of the output variable on the high-dimensional control variable. The specific functional form $\hat{m}\left(X_{\mathrm{it}}\right)$ can be obtained through the use of machine learning algorithms. $V_{\mathrm{it}}$ is the error term and satisfies the assumption of having a zero mean. Using Equations (5) and (6), a three-step method can provide an unbiased estimate of $\theta_{2}$ .

Since the dual machine model allows for the inclusion of more control variables, we include individual fixed effects in the model to further exclude the confounding influence of individual characteristics on health. We use the ridge regression algorithm for estimation with a sample split ratio of 1:4. The estimation results for dual machine learning are reported in Table 4 Model 1. The coefficient on digital governance remains consistent with the baseline results and improves the health of rural residents at a significant level of 1%. Therefore, Hypothesis H1 is once again supported.

**Table 4:** Endogenous treatments.

Variables	Dual machine learning models		CMP methodology	
	Standard model	IV model	Auxiliary equation	Master equation
	Model 1	Model 2	Model 3	Model 4
RDG	0.095^***^ (0.031)	0.165^***^ (0.059)		0.184^***^ (0.054)
IV_DAVC			1.492^***^ (0.053)	
Individual effect	YES	YES	NO	NO
Controls	YES	YES	YES	YES
Region	YES	YES	YES	YES
atanhrho	—	—	−0.098^**^	—
Obs	2,292	2,292	2,292	2,292

The symbols *** and ** indicate significance levels of 1, 5, and 10%, respectively. Clustering-robust standard errors are shown in parentheses. Robust standard errors are shown in parentheses for Models 1 and 2, and cluster robust standard errors are shown in parentheses for Models 3 and 4.

Second, while the dual machine learning model theoretically rules out endogenous interference, there may still be endogeneity due to omitted variables. Therefore, we refer to the studies by Chernozhukov (52) to reconstruct the instrumental variable model of dual machine learning.

(7) $$\mathrm{Healt}h_{i}=\theta_{3}RDG_{i}+g\left(X_{\mathrm{it}}\right)+U_{\mathrm{it}}$$

(8) $$IV_{i}=m\left(X_{i}\right)+V_{i}$$

Equations (7) and (8) use *IV_i_* as the instrumental variable for estimating the coefficients. The remaining variables are consistent with the above.

To complete the estimation, this study uses the Digital Attitude of Village Cadres (DAVC) as the instrumental variable. The CLES village questionnaire surveyed village officials on their attitudes toward digital development. One of the questions asked was whether respondents believed that promoting the integration and sharing of basic agricultural and rural data was necessary. The attitudes of village cadres were categorized into five levels, with “not necessary at all” at the lowest level and “very necessary” at the highest level. This question explored village cadres’ fundamental understanding of digital transformation and attitudes toward rural digital transformation. This study employs the data as a measure of village cadres’ attitudes toward digitalization. The instrumental variable was chosen for the following reasons. First, village cadres in China are both leaders of villages and implementers of higher-level policies, and they play a crucial role in village development (56), including, inevitably, that of digital governance. Second, digitalization requires leaders with sufficient knowledge and skills (57). According to theories of cognition-behaviorism, positive attitudes toward digital development among village cadres can accelerate the digitalization of village governance. Conversely, negative attitudes can impede this process, resulting in a lower level of digital governance. Village cadres’ attitude toward digitalization is related to the level of digital governance in villages. In summary, the correlation of the instrumental variables can be met by the digitization attitude of the village cadres. On the other hand, these attitudes of individual village cadres do not affect residents’ health, thus meeting the exogenous nature of the instrumental variable.

After selecting the instrumental variables, we test their validity. The nonidentifiable hypothesis is rejected because the Kleibergen–Paap RK LM value of 219.851 exceeds 1% significance. Similarly, the weak instrumental variable hypothesis is rejected, as the Kleibergen–Paap rk Wald F statistic of 1007.674 far exceeds the threshold value of 16.38. The results suggest that the instrument variable is valid.

After ensuring that the instrumental variables were valid, we launched further estimation. The results of the instrumental variables model with dual machine learning are presented in Table 4 Model 2. The findings indicate that the coefficient of digital governance improves to 0.165 and exceeds 1% significance, suggesting that digital governance still significantly enhances the health of the rural inhabitants after endogeneity concerns are addressed.

Finally, we use the CMP method again for instrumental variable estimation, as in related studies (21). The conditional mixed process method (CMP) is a common approach for estimating instrumental variables when dealing with ordered discrete data. Table 4 presents the CMP estimation results. Model 3 shows that the regression coefficients of the instrumental variables in the auxiliary equations are positively significant at the 1% degree after controlling for relevant variables. This suggests that the village cadres’ attitude toward digitalization (DAVC) significantly enhances digital governance. Therefore, the instrumental variables are strongly correlated with the endogenous variables. In addition, the statistic atanhrho of CMP is meaningful at the 5% level, which suggests that digital governance is an endogeneity variable and that the CMP model is valid. Model 4 demonstrates that the estimates for digital governance remain significantly positive even after addressing endogeneity, with substantially higher estimated coefficients. This suggests that the baseline results above underestimate the effect of digital governance on the health, and that the CMP estimates are more valid. Hypothesis H1 is once again validated.

#### 4.3.2 Testing of substitution variables

To ensure robust results and avoid the effects of measurement bias, this paper measures some variables with replacement. The first step is to replace the individual health level of the population (health) with the average health level of the household (Health_f). Next, we replace the explanatory variables by remeasuring digital governance and categorizing it into four levels (RDG_r). Finally, this paper replaces household income with respondents’ subjective household economic status and adds some control variables. For the geographic location of the village, the distance from the village to the county town and the elevation of the village are used instead of distance to the township and topographic features (TF). In terms of village health facilities, the number of health rooms and clinics is used instead of the number of garbage cans *per capita*. The distance from the village to the district hospital is also considered. The results of Models 1 through 3 demonstrate that digital governance continues to significantly enhance the health of rural inhabitants, even after the variables are replaced. This confirms the robustness of the previous results.

#### 4.3.3 Testing the change model

In this paper, we replace the ordered probit model with an ordered logit model for validation purposes. Table 5 Model 4 shows the estimation test of the model of ordered logit. The *p*-value in the parallel trend test is 1.000, which is much higher than 0.1, indicating that the model is valid. The results indicate that digital governance continues to significantly enhance the health of rural inhabitants. Second, the Health_f is typically a continuous variable and is estimated using an OLS model to avoid excessive categorization. The results, displayed in Model 5, demonstrate that digital governance continues to significantly enhance the health of rural residents. Based on these findings, the conclusions of this paper can be deemed reliable.

**Table 5:** Robustness tests.

Variables	Substitution of variables			Ordered logit	Reg
	Health_f	Health	Health	Health	Health_f
	Model 1	Model 2	Model 3	Model 4	Model 5
RDG	0.107^***^ (0.033)		0.079^**^ (0.039)	0.136^**^ (0.057)	0.081^***^ (0.025)
RDG_r		0.054^**^ (0.024)			
Controls	YES	YES	YES	YES	YES
Region	YES	YES	YES	YES	YES
Log likelihood	−6075.623	−2776.670	−2730.149	−2772.359	—
Wald	266.28^***^	365.02^***^	438.18^***^	353.95^***^	—
Pseudo R^2^	0.023	0.064	0.079	0.065	0.113
Obs	2,292	2,292	2,292	2,292	2,292

The symbols *** and ** indicate significance levels of 1, 5, and 10%, respectively. Clustering-robust standard errors are shown in parentheses.

### 4.4 Analysis of expandability

In this section, we further explore the effect of digital governance on public health aspects such as longevity and mental health. OLS is used for estimation in this section since the longevity and mental health data are continuous variables. The model’s explanatory and control variables remain the same as above, and Table 6 shows the regression output. Model 1 reveals that digital governance significantly increases the average age of death in villages at the 1% level. This suggests that digital governance is also effective in increasing rural life expectancy and indirectly demonstrates its health benefits. The output of Model 2 reveals that the regression coefficient of digital governance is positive, but it fails the test of significance. Therefore, the influence of digital governance on the mental health of rural residents is considered insignificant. Combined with the aforementioned conclusions, it is evident that current digital governance has primarily improved the physical health of rural residents. However, there is insufficient evidence to support its impact on mental health.

**Table 6:** Analysis of expandability.

Variables	Reg	Reg
	Longevity	Mhealth
	Model 1	Model 2
RDG	3.668^***^ (0.404)	0.041 (0.070)
Controls	YES	YES
Region	YES	YES
R^2^	0.220	0.036
Obs	2,292	2,292

The symbols ***, **, and * indicate significance levels of 1, 5, and 10%, respectively. Clustering-robust standard errors are shown in parentheses.

## 5 Conclusions and recommendations

The concept of rural digital governance has been identified as having a multitude of beneficial effects. This paper presents new insights and empirical evidence on the impact of this governance on health. The present study employs 2021 CLES data from Jiangsu, China, to examine the effects of digital governance on the health, longevity, and mental health of rural residents. It can be concluded that digital governance has a positive impact on rural health. A mechanistic analysis indicates that digital governance can enhance the health of rural residents by improving environmental governance and increasing access to information. Furthermore, our findings indicate that this factor contributes to the longevity of rural residents, yet has no significant effect on their mental health.

This paper presents three policy recommendations based on the aforementioned conclusions.

Firstly, it is imperative that rural digital governance is continuously reinforced in order to more effectively support the health of residents. It is therefore recommended that digital rural policies be reinforced. Furthermore, the beneficial function of digital governance should be enhanced in order to provide improved healthcare services for rural groups. The issue of insufficient supply of rural digital infrastructure remains a significant challenge, which may impede the effectiveness of digital governance. Consequently, it is of paramount importance to reinforce the policy framework to direct digital resources towards rural areas.

Secondly, the full potential of digital governance in environmental governance and access to information should be exploited in order to enhance rural public health. In the context of environmental governance, it is crucial to strengthen the integration between digital platforms and intelligent environmental monitoring equipment, thereby optimising the potential of digital governance. Furthermore, digital platforms should be employed to facilitate collaboration across different levels and areas of government, and to promote the involvement of a diverse range of stakeholders, particularly in rural areas. With regard to access to information, it is essential to expand the number of available channels, increase the quantity of information, and enhance the digital literacy of rural residents. This will result in more efficient digital governance.

Finally, the role of digital governance in mental health should be enhanced by expanding health services. The study results indicate that the impact of digital governance on rural mental health is not significant. While psychological counseling through digital platforms has been common and effective, digital mental health services for rural residents should be enhanced to provide more health benefits.

Admittedly, this research has limitations. It is limited by data availability and uses only cross-sectional data to illustrate the effect on health of digital governance. As a result, the analysis lacks depth and fails to explore dynamic impacts. Additionally, due to the limited number of indicators in the survey data, there are inevitably some shortcomings in the measurement of variables. For instance, the measurement of digital governance is mostly based on the construction of platforms, without quantifying their actual efficacy in a more detailed manner. The limited information available on residents’ health has an impact on this study’s examination of health indicators and behaviors, which in turn restricts the depth of analysis. These shortcomings can be addressed in future research.

## Data availability statement

The original contributions presented in the study are included in the article/supplementary material, further inquiries can be directed to the corresponding author.

## Ethics statement

Ethical review and approval was not required for the study of human participants in accordance with the local legislation and institutional requirements. Written informed consent from the participants was not required to participate in this study in accordance with the national legislation and the institutional requirements.

## Author contributions

YC: Conceptualization, Data curation, Investigation, Methodology, Software, Validation, Writing – original draft, Writing – review & editing. QY: Data curation, Funding acquisition, Investigation, Methodology, Supervision, Writing – original draft, Writing – review & editing.
